# Iloprost therapy achieves good clinical and radiological short and mid-term outcomes in patients with idiopathic aseptic osteonecrosis of the knee joint also in ARCO level II

**DOI:** 10.1007/s00402-025-06057-7

**Published:** 2025-10-10

**Authors:** Stephanie Kirschbaum, Carsten Perka, Moses El-Kayali, Clemens Gwinner, Thula Cannon Walter-Rittel, Maurice Soujon, Stefanie Donner

**Affiliations:** 1 Center for Musculoskeletal Surgery, Charité - University Medicine Berlin, Berlin, Germany; 2 Department of Radiology, Charité - University Medicine Berlin, Berlin, Germany

**Keywords:** Aseptic osteonecrosis, Bone marrow oedema, SONK, Iloprost

## Abstract

**Aims:**

The aim of this retrospective study was the evaluation of the patient-reported and radiological outcome of intravenous Iloprost therapy in the treatment of spontaneous osteonecrosis of the knee (SONK).

**Methods:**

36 patients (age 57.3 ± 8.7 years, 38.9% women, 61.1% men) who received Iloprost between 2018 and 2021 due to SONK (ARCO I and II) were included in this retrospective cohort study. Outcome was evaluated by pre- and postinterventional pain (Numeric Rating Scale - NRS), patient reported outcome (subjective knee value (SKV), Oxford Knee Score (OKS)) at latest follow-up (2.9 months ± 1) as well as quantitative artificial intelligence assisted analysis of bone marrow edema (BME) in Magnetic Resonance Imaging (MRI) before and after 3 months.

**Results:**

Radiologically, there was a 71% reduction in edema (pre-intervention: 37.0 cm³±37.7, post-intervention: 10.8 cm³ ± 14.9, *p* < 0.01). Overall satisfaction was 2.0 ± 1.3, SKV was 83.3%±16.6 and NRS at follow-up was 1.3 ± 1.8. OKS reached 33.6 ± 12.0. No major complications were observed. Rare side effects were dizziness which required premature termination of Ilomedin therapy on day 3.

**Conclusion:**

Iloprost treatment seems a safe and promising therapeutic option also in SONK with excellent subjective outcome and reduction of BME of 70% within 3 months after Iloprost infusion.

**Supplementary Information:**

The online version contains supplementary material available at 10.1007/s00402-025-06057-7.

## Introduction

Aseptic osteonecrosis (AON) of the knee joint is a painful disease with a prevalence of 1.4–9.5% depending on age and comorbidities [1, 2, 3]. Mechanical and ischemic etiology of AON is controversially discussed. Previous studies stated that microfractures of the trabecular bone based on bone density reduction can lead to bone marrow edema (BME) and finally to osteonecrosis [4, 5]. Other studies highlighted the fact that insufficiency fractures look different than AON on MRI and should therefore classified separately [6]. An alternative hypothesis is an ischaemic process leading to increased intraosseous pressure and decreased venous outflow, ultimately resulting in bone marrow oedema (BME) [7]. Therefore, some authors describe “idiopathic” BME as a precursor of avascular necrosis [8, 9, 10, 11] resulting in an inconsistent use of the term “spontaneous osteonecrosis of the knee” (SONK) and “BME-syndrome” for any kind of idiopathic BME in Association Research Circulation Osseous (ARCO) stage I and II [12, 13, 14]. Furthermore, there are no guidelines for the treatment of SONK, regardless of classification. The recommendations for conservative treatment are very inconsistent and range from simple relief of the knee joint, the use of non-steroidal anti-inflammatory drugs (NSAIDs), vitamin C and calcium to the off-label use of drugs such as bisphosphonates or prostacyclins [1, 2, 15, 16, 17, 18]. While use of bisphosphonates should prevent progressive bone resorption [1, 2, 16] prostacyclin (Iloprost) as vasoactive, platelet aggregation inhibiting prostaglandin I2 analogue should improve bone perfusion [1, 17, 18]. While there were good results demonstrated for conservative Iloprost therapy in early stages (ARCO I and II) of aseptic femoral head necrosis [19, 20, 21], hardly any data exist regarding the clinical and/or radiological evaluation of Iloprost therapy for SONK [18]. Therefore, the aim of this study was the subjective, clinical and radiological evaluation of Iloprost infusion therapy for the treatment of SONK.

## Methods

The retrospective study design was approved by the local ethics board (EA4/251/21).

### General confirmation of diagnosis and treatment

The diagnosis of SONK was confirmed by (1) medical history, (2) clinical symptoms and (3) x-rays as well as magnetic resonance imaging (MRI) in all cases. According to the ARCO classification of 2019, visible BME in MRI without pathological signs in CT or radiography were assessed as ARCO stage 1, and visible BME in MRI with signs of sclerosis in CT and minor osteolysis and signs of sclerosis in radiography were assessed as ARCO stage 2 [22]. In every case demonstrating ARCO stage I (*n* = 7) and II (*n* = 29), all conservative therapeutic options such as partial weight bearing -with or without the use of vitamin D and calcium were already tried for at least 3 months without any symptom relief. Option of off-label- use of Iloprost was discussed individually with each patient. Patients with a history of thromboembolic events, coronary vascular disease, known aneurysms or difficult-to-control arterial hypertension were not advised to receive Iloprost infusion therapy. Patients with previous fractures or necrotic zones on conventional x-ray (ARCO IIIa/b) or osteoarthritis (ARCO IV) were not suitable for conservative therapy options and therefore treated by other means (e.g. surgical decompression, osteochondral autologous transplantation, unicondylar knee replacement).

**Fig. 1:**
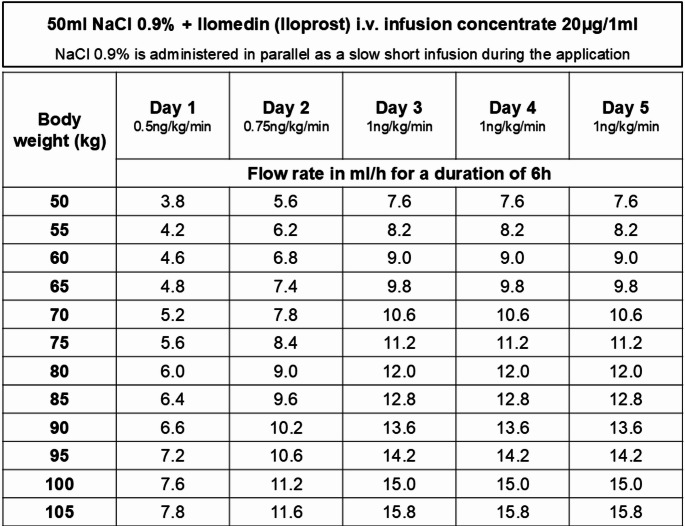
Iloprost therapy plan

In case patients decided for an Iloprost therapy it was administered as a 5-day, infusion therapy as previously described and published by Röhner et al. [17] (Fig. 1). Partial weight bearing with 2 forearm crutches was recommended to the patients for a duration of 6 weeks. After 3 months, every patient received a standard follow-up visit in our outpatient department as well as an MRI of the knee. Assessment of PROMs in contrast was not part of standard protocol at the three months follow-up.

### Patient enrollment

For this retrospective cohort study, all patients admitted to our department between 01/01/2018 and 05/31/2021 with an International Statistical Classification of Diseases and Related Health Problems (ICD) code for “idiopathic aseptic bone necrosis” were identified in the hospital internal information system and screened regarding inclusion and exclusion criteria.

**Fig. 2:**
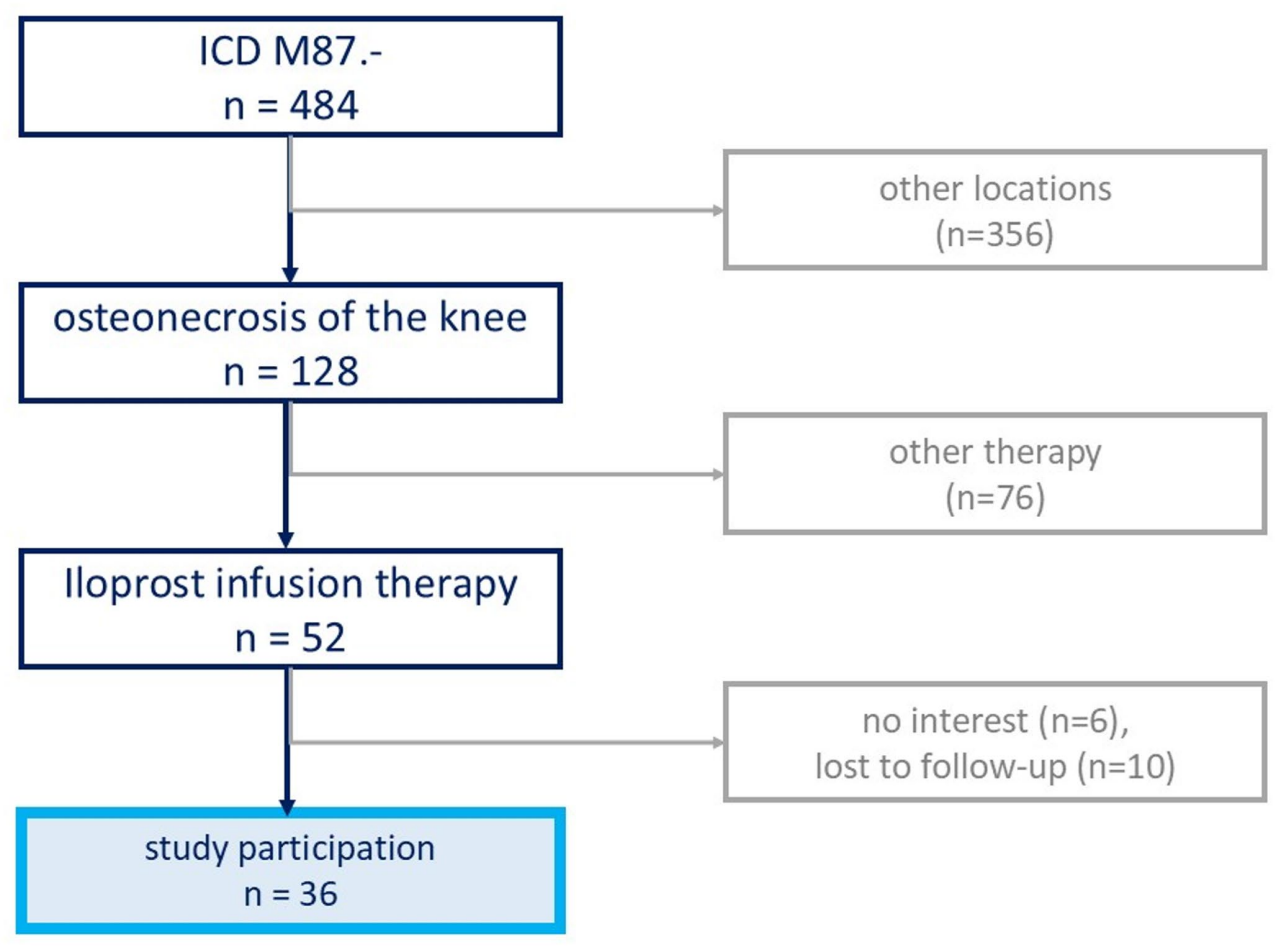
patients’ enrolment

Inclusion criteria were: SONK of the knee joint and treatment by inpatient intravenous Iloprost therapy due to failed conservative outpatient treatment. Osteonecrosis in other locations, such as femoral head necrosis or humeral head necrosis, secondary osteonecrosis of the knee due to steroid medication, alcohol addiction, chemotherapy, trauma, or osteoarthritis, as well as other forms of therapy than intravenous Iloprost therapy were excluded (Fig. 2). All remaining 52 patients were informed about the study. Thirty-six out of these 52 patients participated in the study (14 female (38.9%) and 21 male (61.1%). Informed and written consent was obtained from all patients.

### Patient demography and patient reported outcome measurement

Age, sex, Body Mass Index (BMI), number of comorbidities as well as the clinical examination results, pain level (Numeric Rating Scale – NRS) at time of diagnosis and follow-up were taken from the patient’s record. In addition, all study participants completed the Subjective Knee Value (SKV), the Oxford Knee Score (OKS) as well as pain level in rest and activity (NRS) at the study related follow-up. Satisfaction with the therapy outcome was evaluated by the patients according to the countries school grading system (a Likert scale ranging from 1 to 6, grade 1–6, 1 = very good, 6 = insufficient).

### Radiological outcome

**Fig. 3:**
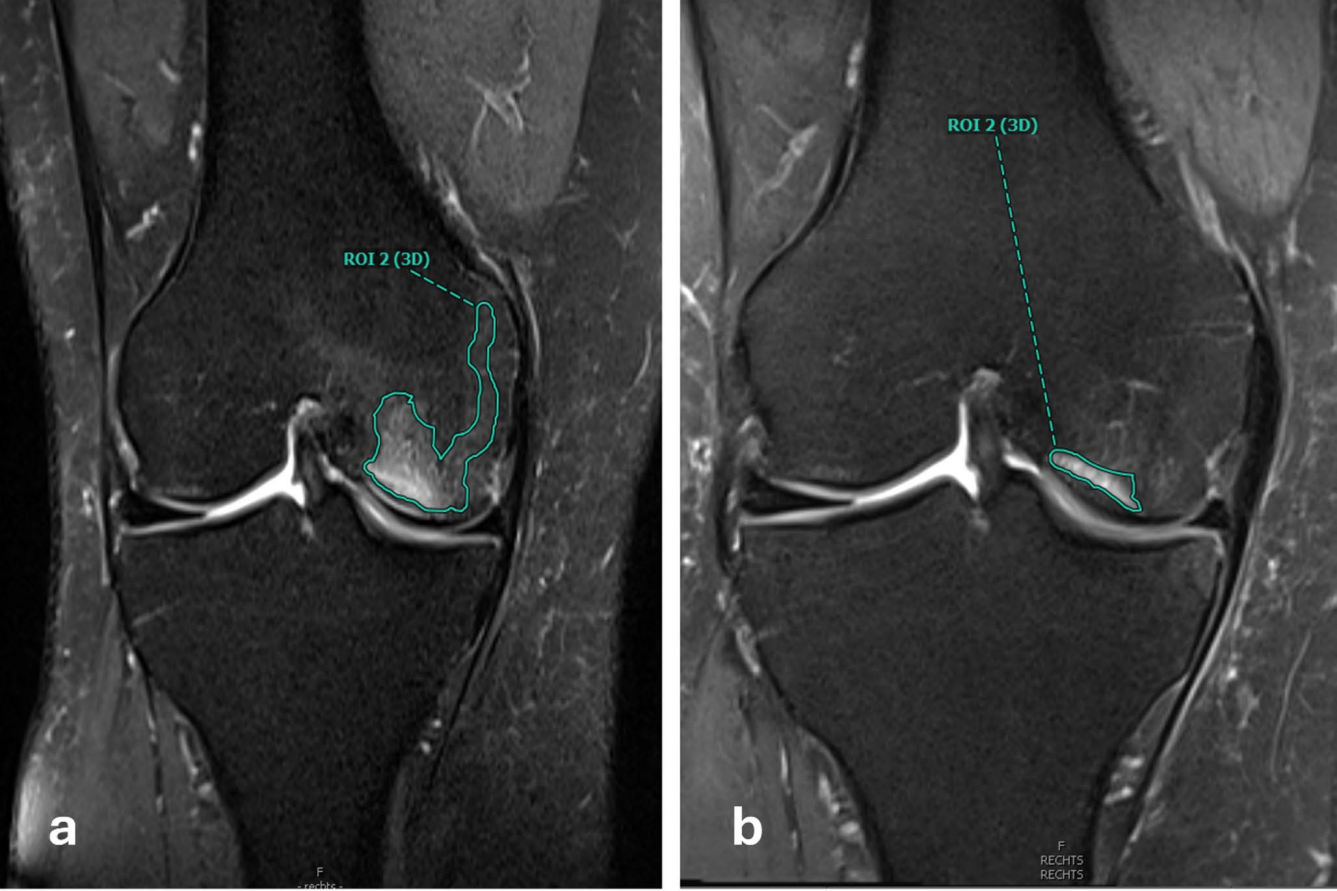
(a) of region of interest (ROI) of observer 1 in pre-treatment MRI (PD fs, coronal slicing, 3 mm thickness), (b) ROI of observer 1 in 3 months post treatment MRI (PD fs, coronal slicing, 3 mm thickness)

The radiological outcome was assessed by manual volume measurements of the bone marrow edema in the pre-treatment and follow-up MRI at the 3 months control visit (Fig. 3). Two independent readers measured BME in all patients at baseline and follow-up.

**Fig. 4:**
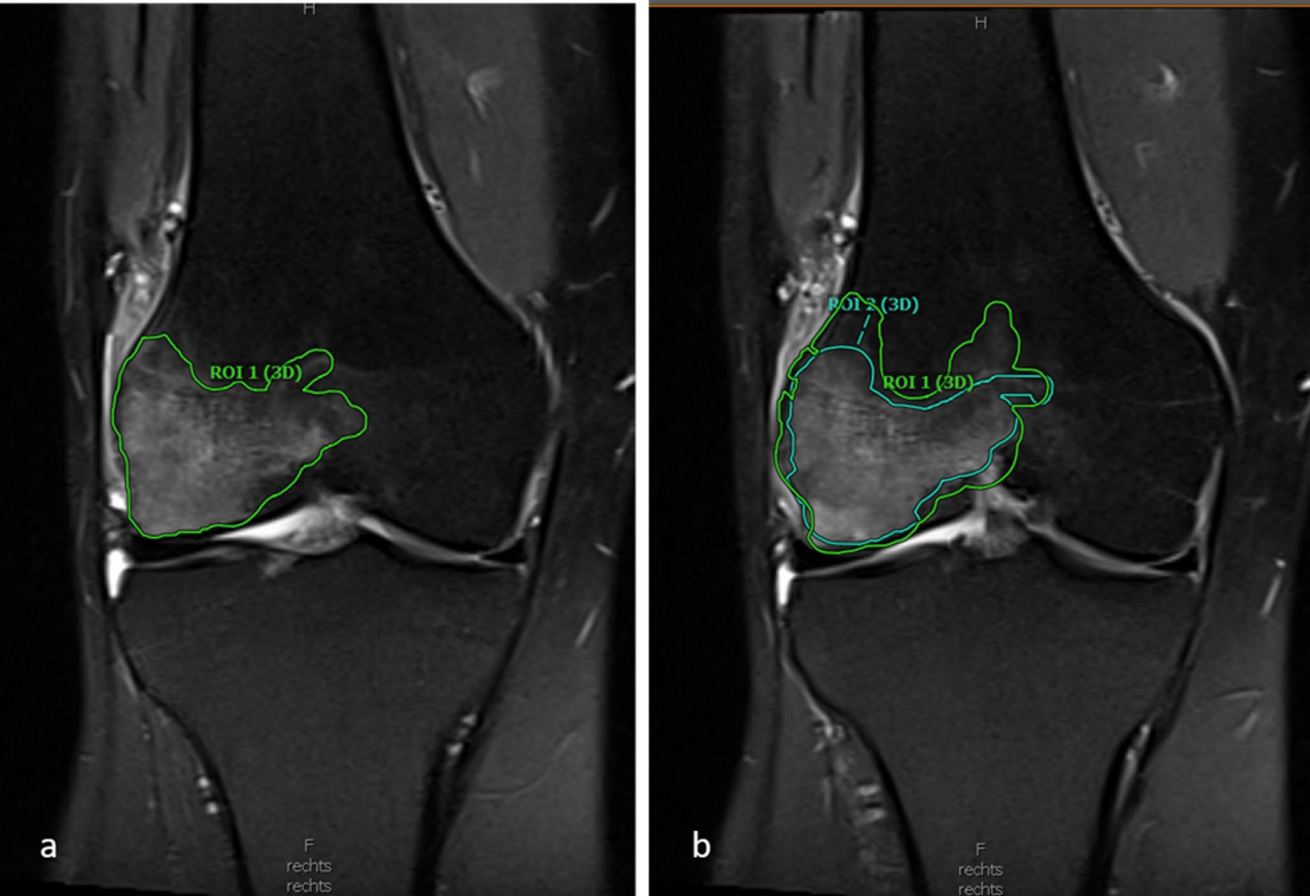
(a) of region of interest (ROI) of reader 1 in pre-treatment MRI (PD fs, coronal slicing, 3 mm thickness); (b) comparison of ROI delineation between observer 1 and 2, which used different threshold signal intensities to determine delineation

Measurements were made using the free-hand 3D region of interest (ROI) tool in the Visage PACS [23] viewer (Visage Imaging, Version 7, Berlin, Germany). BME was contoured in consecutive image slices on coronal proton density fat saturated (PD fs) sequences. The software identified the contours of BME semi-automatically and was manually corrected and adjusted where necessary (Fig. 4).

**Fig. 5:**
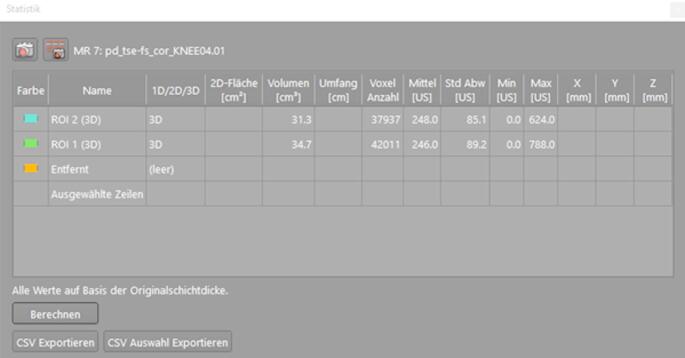
3D volume for segmented ROIs for reader 1 (green delineation, green ROI) and reader 2 (blue delineation, blue ROI)

For each measurement, the tool then calculated the contoured volume (Fig. 5). Due to the retrospective study design, not all MRIs were conducted in-house. Therefore, sequence parameters, slice thickness and scanner type differ between patients. However, a PD fs sequence, which depicts BME, is a standard sequence in most knee MRI protocols. Wherever available, coronal PD fs sequences were segmented, as a default, sagittal were segmented, where coronal PDfs sequences were not available. Slice thickness in pre-interventional MRI was 3.2 mm ± 0.4 [2.5-4.0] and 3.1 mm ± 0.4 [2.5–4.5] in post-interventional MRI.

### Statistical analysis

Statistical analysis was performed using Statistical Package für Social Sciences Version 26 (SPSS Inc., Chicago, IL, USA). Data are expressed as the mean ± SD as well as range. The Shapiro‒Wilk test was used to test the Gaussian distribution. Comparison of the pain level and volume of the bone marrow edema was performed using the Wilcoxon-Test. Inter-rater reliability was tested using Cronbach α analysis to evaluate the intra class correlation (ICC). The ICC was interpreted using the categorization by Landis and Koch (≤ 0.20, slight; 0.21–0.40, fair; 0.41–0.60, moderate; 0.61–0.80, substantial; and ≥ 0.81, almost perfect) [24]. The significance level of all tests was 5%. Furthermore, statistical correlations between the percentage reduction in bone marrow edema and outcome parameters—including OKS, pain levels at rest and during activity, SKV, and patient satisfaction—were analyzed using Pearson’s correlation coefficient.

## Results

Mean patient age was 57.3 ± 8.7 [40–75] years with average BMI of 27.0 ± 4.5 [17.9–39.1] kg/m^2^. The majority of patients (61.1%) had one comorbidity (0 = 33.3%, 1 = 27.8%), 22.2% had two, and 16.7% of patients had three or more comorbidities. The mean time between Iloprost-infusion and follow-up MRI was 2.9 ± 1 [1.3–5.5] months. The mean clinical follow-up between Iloprost infusion and last assessment was 27.2 ± 14.3 [2.4–54.1] months.

In one case, Iloprost therapy was discontinued on day 3 due to dizziness. No other or severe side effects that required a discontinuation of Iloprost therapy were noted in this study.

### Patient reported outcome

The mean pain level before Iloprost therapy was 4.8 ± 1.4 [3–9]. At clinical Follow-up average pain level at rest was 1.3 ± 1.7 [0–8] and 3.0 ± 2.4 [0–10] while activity which was both significant less intense (Wilcoxon < 0.01). An overview about level of patient reported outcome scores is demonstrated in Table 1.

**Table 1:** PROMS at follow-up assessment

PROMS	Score (mean + / - SD; Range)
Subjective Knee Value [%]	83.3 ± 16.6 [40–100]
Oxford Knee Score [points]	33.6 ± 12.0 [14–48]

*Patient reported outcome

**Fig. 6:**
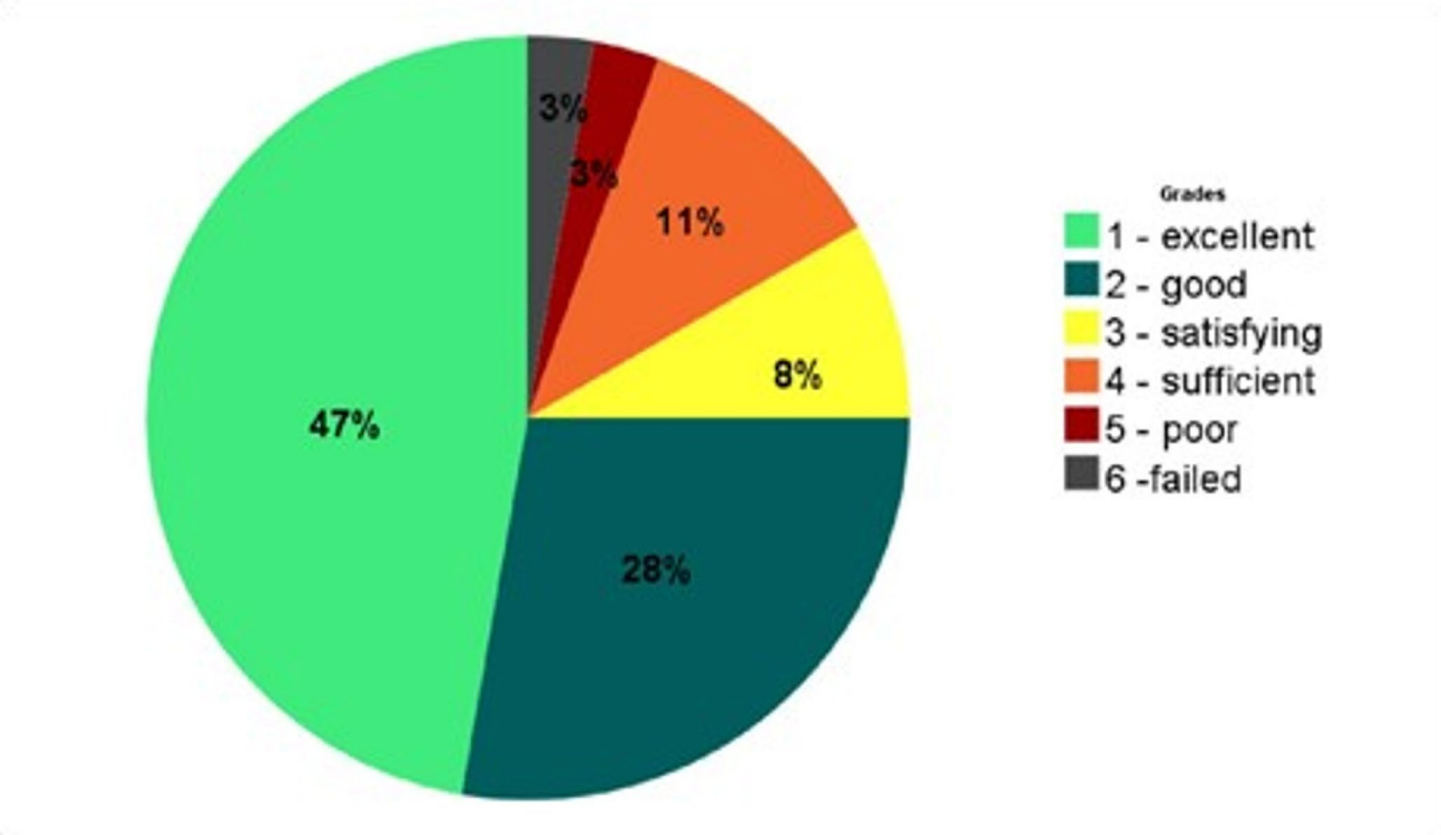
Distribution of satisfaction after Iloprost therapy at latest follow-up according to German school grading system (1-excellent, 6- failed)

Overall satisfaction was rated 2.0 ± 1.3 [1–6]. Distribution of grades is demonstrated in Fig. 6.

### Radiological outcome

**Fig. 7:**
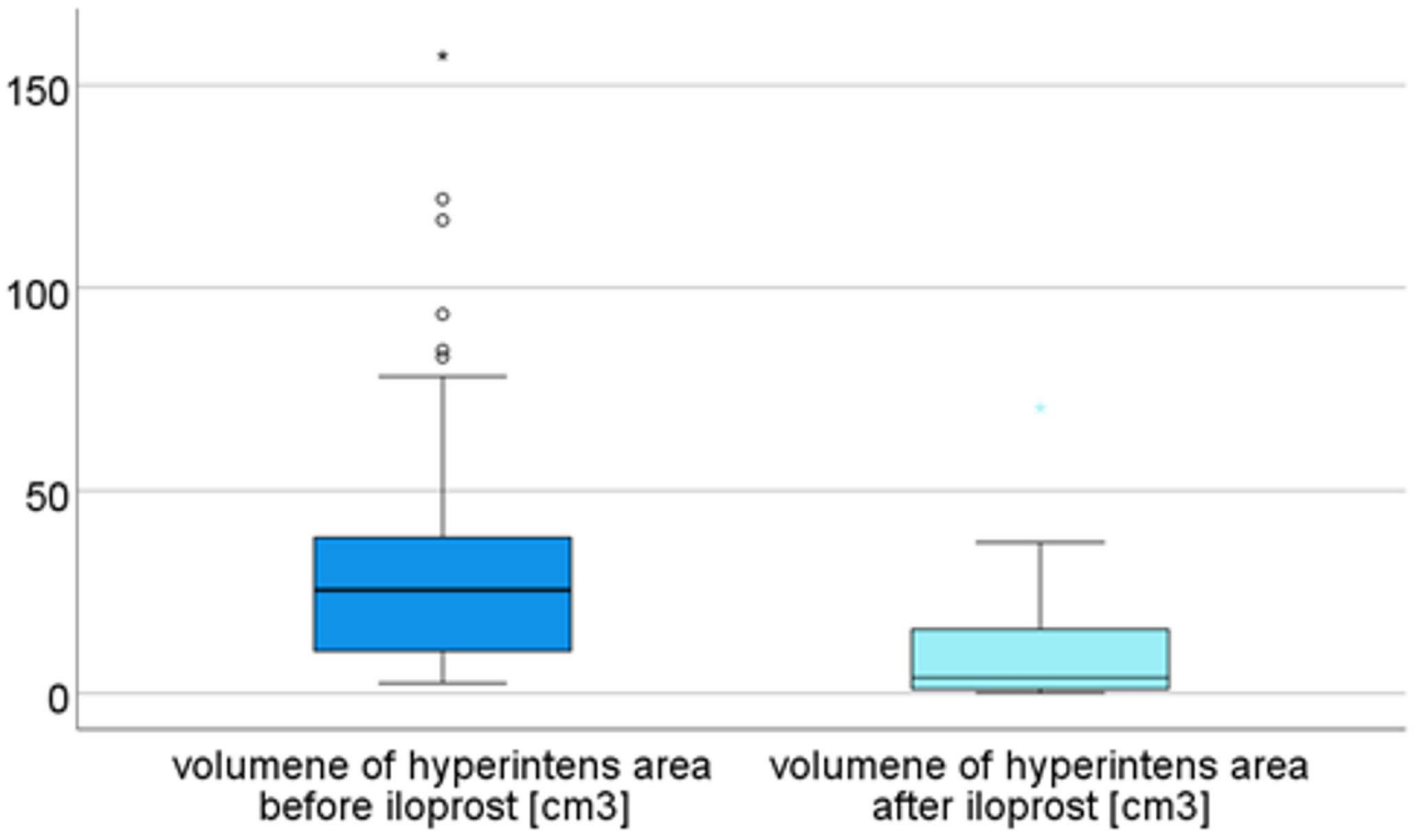
Volume of hyperintense area in MRI before and after Iloprost therapy showed significant reduction (*p* < 0.001)

Average volume of hyperintense area was 37.0 cm³ ± 37.7 [2.5-157.3] before and 10.8 cm³ ± 14.9 [0.0-70.4] after Iloprost therapy (*p* < 0.01, Wilcoxon-test). Average reduction of hyperintensity, i.e. BME was 70.7%. ICC was 0.98 [95% CI 0.966–0.989, *p* < 0.01] (Fig. 7).

There were no significant correlations between level of BME reduction and OKS, NRS level at rest or subjective knee value as outcome parameters (each *p* > 0,272). In contrast, NRS level while activity (correlation − 0.41, *p* = 0.014) and patients’ satisfaction (correlation − 0.38, *p* = 0.021) showed moderate but significant correlation.

## Discussion

Up to now, only a few, small, extremely heterogeneously designed studies have examined the effect of Iloprost on “BME syndrome”/ SONK of the knee, with cohorts of up to 31 knees and short-term follow-up of no more than one year [1, 18, 20, 25, 26, 27]. Notably, both terms have been used inconsistently in the literature, making it even more difficult to evaluate existing data as a possible self-limiting condition is compared to an actual progressive osteonecrosis. However, the recent study presents the most homogeneous and, to date, the largest cohort evaluating the effect of off-label Iloprost infusion therapy in the treatment of SONK, conducted without any industry support. Furthermore, this is the first study to assess outcomes not only through subjective measures and patient-reported outcome measures (PROMs) at a mid-term follow-up of 27 months but also by quantitatively measuring the reduction in the extent of bone marrow edema on MRI.

### Clinical and radiologic outcome

Recent study demonstrated significant reduction of pain (by ca. 60%) and satisfying PROM 2.3 years without major complication after intravenous Iloprost infusion due to SONK (ARCO I&II). Interestingly, some authors even reported immediate pain relieve after first infused dose of Iloprost in up to 80% of the patients [1, 20] with ongoing pain decrease until last follow-up [1, 25, 27]. Assuming an ischemic etiology this might be caused by either the vasodilatation and therefore improved perfusion or the anti- inflammatory effect of Iloprost [28]. Improved perfusion also seems to support bony regeneration as BME volume decreased by ca. 70% within 3 months after intravenous application in the recent study. Interestingly, Iloprost shows significant less reduction of BME if it is associated not to trauma or idiopathic reasons but to degenerative changes [29]. It therefore seems plausible to assume an ischemic genesis. However, most existing studies only evaluate – if they do at all – appearance of BME in MRI by comparing pre- and postinterventional ARCO grade [20] or describing the change in MRI barely distincting between “remission/decrease and increase” [1, 18, 26]. Notably, no fixed criteria were defined to determine a de- or increase in bone marrow edema. Instead, that classification (“remission/decrease/increase”) relies only on the subjective assessment of the observers, resulting in limited inter-observer reliability and transparency. Moreover, the intra-individual follow-up time of the post-interventional MRI of these studies varies between 3 months and 1 year reducing their significance considerably [1, 26]. Furthermore, some of the studies can provide follow-up MRI data only of a part (65–85%) of their cohort which significantly reduces the quality and validity of the existing literature [1, 26]. The only other existing study performing a quantitative MRI analysis is funded and supported by Bayer Schering Pharma AG itself [25]. With this exception, no other study analyzed quantitatively the extend of BME before and after Iloprost therapy in such a detailed manner of the complete investigated cohort. Surely, this represents a strength of the recent study.

It is noteworthy that only pain levels during activity and overall patient satisfaction showed a moderate correlation with the extent of BME reduction. Naturally, potential bias must be considered, as the MRI evaluation was performed at the 3-month follow-up, whereas pain levels and satisfaction were assessed only at the study-related final follow-up after 27 months. However, since overall satisfaction appears to be less dependent on the exact timing of follow-up, the degree of BME reduction may serve as a promising indicator for long-term outcomes. Further, more detailed analysis of BME—ideally within the framework of prospectively designed studies—could help to validate and expand upon this observation.

### Limitations and strengths

As already mentioned, the retrospective study design itself surely represents the biggest limitation of the recent study. First, we acknowledge the possibility of selection bias. However, to avoid debate over treating a potentially self-limiting condition such as “bone marrow edema syndrome”, we included only patients who had failed conservative treatment for at least three months and reported increasing pain. Additionally, MRI had to show severe edema with early osteonecrotic changes, typically corresponding to ARCO stage II. We believe these criteria help minimize potential selection bias, particularly regarding the treatment of a possibly self-limiting condition. Second, due to retrospective design, some available MRI PD fs sequences differ concerning the way (coronal, sagittal, axial) and thickness of slicing. Second, due to the retrospective design, some of the available MRI PD fat-saturated sequences differed in imaging plane (coronal, sagittal, or axial) and slice thickness. However, since slice thickness did not show any significant difference between pre- and post-interventional MRI (*p* = 0.121), this factor does not appear to introduce relevant bias. In addition, in all but two cases, observers used the same imaging plane for each patient before and after Iloprost therapy. As the majority of cases thus underwent individual pre- and post-interventional comparisons without changes in key imaging parameters, any potential bias from this source should be minimal. Moreover, the detailed, software-assisted 3D volume calculation—based on consecutively marked ROIs in calibrated MRI and adjusted for slice thickness—provides novel data. Third, the study lacks a control group due to retrospective study design. Bisphosphonates were not used by authors group, as their side effects—especially severe esophagitis and, less commonly, osteonecrosis of the jaw—are reported to be more frequent and serious than those of Iloprost (e.g., headaches, dizziness, blood pressure issues) [Quelle]. Core decompression, usually combined with Iloprost, is reserved for ARCO stage III SONK at authors department. However, with only four patients treated this way during the above-mentioned follow-up period, this group does not offer a meaningful comparison. We attempted to set up a prospective study comparing Iloprost with conservative treatment (partial weight bearing, NSAIDs, Vitamin D), but this design required a formal drug approval study due to Iloprost’s off-label use—something not financially feasible for our research group. So unfortunately, off-label-use is complicating a prospective randomized study design.

However, the present study also has several strengths beyond the detailed BME analysis mentioned above. Another strength surely is the homogenous patient cohort due to very strict inclusion and exclusion criteria. Jäger et al. included 34 knees with mid-term follow-up (33 months), but treated some of them with additional core decompression, thus reducing the conclusiveness of the Iloprost therapy results [20]. Aigner et al. too, treated a part of the cohort with additional core decompression without any further sub analysis of those patients [27]. Meizer et al. for example included a total of 50 knees but different kinds of BME (8 idiopathic, 10 posttraumatic, 32 secondary due to degenerative changes) [29]. Mayerhoefer et al. treated patient by oral Iloprost medication and changed in unsuccessful cases to intravenous application without any further distinction regarding result presentation [26]. Another strength of the recent study is the relatively long follow-up of 2.3 years demonstrating at least mid-term lasting effect of Iloprost therapy. Majority of existing literature only examines follow-up of maximum on year [1, 25, 26, 27]. Only one more recent retrospective study of Zippelius et al. is confirming good clinical mid-term results (33 months) of Iloprost used for therapy of BME of only 15 knees [18].

## Conclusion

Clinical and radiological outcome parameters recent study demonstrated satisfying clinical short- and mid-term outcome, without any severe complications as well as average BME volume decrease of 70% within three months after Iloprost infusion therapy. Therefore, Iloprost treatment seems a safe and promising therapeutic option also in SONK.

## Supplementary Information

Below is the link to the electronic supplementary material.

Supplementary Material 1

## Data Availability

Data is provided within the manuscript and supplementary information files.

## Abbreviations

SONK: Spontaneous osteonecrosis of the knee
NRS: Numeric Rating Scale
SKV: Subjective knee value
OKS: Oxford Knee Score
BME: Bone marrow edema
MRI: Magnetic Resonance Imaging
AON: Aseptic osteonecrosis
ARCO: Association Research Circulation Osseous
NSAIDs: Non-steroidal anti-inflammatory drugs
ICD: International Statistical Classification of Diseases and Related Health Problems
BMI: Body Mass Index
ROI: Region of interest
SSPS: Statistical Package für Social Sciences
OC: Intra class correlation
PROM: Patient-reported outcome measures
