# Supplementary material for: Iloprost therapy achieves good clinical and radiological short and mid-term outcomes in patients with idiopathic aseptic osteonecrosis of the knee joint also in ARCO level II
Source: Arch Orthop Trauma Surg. 2025 Oct 10;145(1):462. doi: 10.1007/s00402-025-06057-7 (PMC12513965; doi:10.1007/s00402-025-06057-7)
Supplement: Supplementary file 1 — Supplementary Material 1 [file 402_2025_6057_MOESM1_ESM.pdf]

No	Sex	Age	Follow_up	FU_PROM	BMI	No previou	amount di	satisfactio	SKV	NRS_prä	NRS_Ruhe	NRS_sport	OKS	V_KMÖ_P	V_KMÖ_P	Volumenre	V_KMÖ_P	V_KMÖ_P	Volumenre	Slice_Thi	Slice_Thickness_MRT_Post
1	1	65	2,20	34,80	21,80	0	2	1	80	6	1,00	5,00	31,00	24,40	4,64	81,00	19,00	0,51	97,30	4,00	4,00
2	2	59	3,20	9,00	23,00	0	0	1	80	4	1,50	2,00	42,00	31,00	10,80	65,20	39,00	25,30	35,10	3,00	3,00
3	2	52	4,30	25,60	26,00	0	0	3	50	8	5,00	5,00	32,00	11,60	0,81	93,00	10,50	0,86	91,80	4,00	3,20
4	2	52	3,10	27,60	25,70	0	0	2	95	5	1,00	1,00	16,00	84,60	35,40	58,20	96,20	39,70	58,70	3,00	3,00
5	2	56	2,10	36,90	27,40	0	2	1	95	4	0,00	1,00	47,00	12,10	1,12	90,70	9,88	1,74	82,40	4,00	4,50
6	2	67	5,30	15,10	32,50	0	1	4	40	4	2,00	5,00	17,00	10,50	9,47	9,80	11,50	9,68	15,80	3,00	3,00
7	1	59	2,90	40,00	22,60	0	3	1	100	4	0,00	0,00	48,00	31,30	0,45	98,60	44,20	6,18	86,00	3,00	3,00
8	1	53	2,30	19,00	26,50	0	3	1	90	5	0,00	2,00	46,00	27,60	0,24	99,10	25,10	0,24	99,10	3,00	3,00
9	2	61	3,10	41,60	27,80	0	1	2	90	5	0,00	2,00	47,00	52,20	5,33	89,80	52,10	14,20	72,70	3,00	3,00
10	2	73	1,30	22,40	25,40	0	2	5	70	4	2,00	5,50	21,00	82,90	0,34	99,60	107,60	23,70	78,00	4,00	2,50
11	2	44	3,20	28,40	25,10	0	0	1	90	4	1,00	2,00	46,00	10,20	1,84	82,00	13,80	1,03	92,50	3,00	3,00
12	2	56	1,80	29,50	27,40	0	5	2	80	6	0,00	4,00	19,00	36,90	21,80	40,90	37,80	20,40	46,00	3,30	3,30
13	1	75	3,10	38,70	20,30	0	0	1	100	3	0,00	0,00	19,00	33,40	1,85	94,50	39,60	13,70	65,40	4,00	4,00
14	2	52	2,20	34,70	30,90	0	1	4	80	4	1,00	5,00	24,00	116,70	15,70	86,50	125,90	24,70	80,40	3,00	3,00
15	2	54	2,40	14,00	30,90	0	1	4	80	3	1,00	5,00	24,00	93,50	70,40	24,70	106,30	75,00	29,40	3,00	3,00
16	2	54	2,50	8,10	30,90	0	1	4	80	4	1,00	5,00	24,00	157,30	27,00	82,80	171,50	40,10	76,60	3,00	3,00
17	1	54	3,40	37,70	26,90	1	1	3	85	9	8,00	8,00	28,50	32,60	24,30	25,50	39,50	29,60	25,10	3,00	2,50
18	1	50	3,00	51,80	37,00	0	1	2	80	4	3,00	3,00	29,00	10,20	1,84	82,00	13,80	1,03	92,50	3,00	3,00
19	1	68	2,30	22,00	27,50	0	3	1	100	4	0,00	0,00	46,00	4,50	0,48	89,30	6,98	2,04	70,80	3,00	2,50
20	1	68	2,70	32,30	27,50	0	3	1	100	3	0,00	0,00	46,00	6,38	1,05	83,50	22,50	2,11	90,60	3,00	3,00
21	2	42	5,50	6,90	27,40	0	0	1	95	4	0,00	2,00	46,00	121,90	37,30	69,40	125,30	46,80	62,60		
22	1	71	2,50	31,80	28,20	1	2	2	98	4	1,00	2,00	45,00	24,70	19,60	20,60	25,80	18,10	29,80	3,00	3,00
23	2	50	2,70	40,00	27,50	0	5	3	50	7	5,00	5,50	28,00	38,50	3,38	91,20	38,00	3,57	90,60	4,00	3,00
24	2	61	1,40	8,80	28,40	0	2	2	40	5	2,00	5,00	30,00	8,33	0,54	93,50	9,56	0,95	90,10	3,00	3,00
25	2	49	2,00	20,40	23,70	0	0	1	90	5	1,00	2,50	44,00	17,40	10,60	39,10	22,40	13,90	37,90	3,50	3,50
26	2	43	4,70	54,10	25,90	0	2	1	100	4	0,00	0,00	48,00	32,30	1,72	94,70	35,90	1,22	96,60	3,00	3,00
27	2	57	2,60	45,30	26,00	1	1	2	85	4	1,00	2,00	43,00	22,80	15,80	30,70	20,70	17,10	17,40	3,00	3,00
28	1	61	2,50	17,10	17,90	0	1	2	80	6	3,00	5,00	26,00	26,30	15,50	41,10	22,40	13,90	37,90	3,00	3,00
29	2	59	4,50	46,10	21,20	1	0	2	80	5	0,00	2,00	45,00	2,93	0,27	90,70	3,32	0,20	94,10	3,00	3,00
30	1	52	4,10	4,80	21,20	1	0	6	65	5	1,00	10,00	41,00	2,54	2,24	11,80	1,69	1,80	-6,50	3,00	3,00
31	2	40	2,70	36,80	39,10	1	2	2	95	4	1,00	2,00	14,00	10,30	1,90	81,60	35,20	6,65	81,10	3,00	3,00
32	1	70	2,50	23,70	33,20	1	1	1	75	3	1,00	3,50	36,00	11,90	0,11	99,10	16,10	1,21	92,50	3,00	3,00
33	2	57	2,50	46,40	28,90	0	0	1	95	4	0,00	3,00	47,00	78,10	30,70	60,70	38,10	19,50	48,80	2,50	3,00
34	1	65	3,60	17,10	21,40	0	2	1	100	6	0,00	0,00	36,00	14,70	4,42	69,90	12,70	1,64	87,10	3,00	3,00
35	2	56	3,00	8,20	28,40	0	0	1	97	6	0,00	0,00	14,00	35,90	9,23	74,30	37,50	10,00	73,30	3,00	3,00
36	1	58	2,00	2,40	31,00	0	0	1	90	7	2,00	3,00	14,00	14,70	0,00	100,00	15,30	0,00	100,00	3,00	3,00
